# Default Mode Connectivity in Youth With Perinatally Acquired HIV

**DOI:** 10.1097/MD.0000000000001417

**Published:** 2015-09-18

**Authors:** Megan M. Herting, Kristina A. Uban, Paige L. Williams, Prapti Gautam, Yanling Huo, Kathleen Malee, Ram Yogev, John Csernansky, Lei Wang, Sharon Nichols, Russell Van Dyke, Elizabeth R. Sowell

**Affiliations:** From the Department of Pediatrics, Children's Hospital Los Angeles, Los Angeles, CA (MMH, KAU, PG, ERS); Department of Biostatistics, Harvard School of Public Health, Boston, MA (PLW, YH); Feinberg School of Medicine, Northwestern University, Chicago, IL (KM, RY, JC, LW); Department of Neurosciences, Division of Pediatric Neurology, University of California San Diego, La Jolla, CA (SN); Tulane University School of Medicine, New Orleans, LA (RVD); and Keck School of Medicine, University of Southern California, Los Angeles, CA, USA (ERS).

## Abstract

Youth with perinatally acquired human immunodeficiency virus (PHIV+) survive longer with combination antiretroviral therapy, but remain at risk for poor cognitive outcomes. We evaluated whether markers of HIV disease severity relate to default mode resting-state functional connectivity in PHIV+ youth.

We conducted resting-state functional neuroimaging and cognitive testing in a subset of 40 PHIV+ youth recruited from a single study site of the Adolescent Master Protocol study conducted by the Pediatric HIV/AIDS Cohort Study (PHACS) network. Current and past HIV disease severity measures (nadir CD4 lymphocyte percentages and peak HIV RNA plasma levels) were obtained from medical charts.

We evaluated associations of both HIV disease severity measures and cognitive functioning with between- and within- default mode network (DMN) connectivity using Analysis of Functional NeuroImaging multiple regression analyses, controlling for multiple comparisons.

Of the 40 youth, 31 (mean age = 16.5 years) with minimal motion during scans were included. We observed global alterations in DMN within- and between-network connectivity, with significant associations between disease severity and DMN BOLD correlations. Furthermore, patterns of connectivity with the posterior cingulate cortex (PCC) and medial prefrontal cortex (mPFC) that varied as a function of peak HIV RNA were found to predict processing speed ability.

Alterations in within- and between-network DMN connectivity in PHIV+ youth may reflect global reorganization of the DMN; this could lead to compensatory alterations in both the within- and between-connectivity of large-scale networks, which may ultimately relate to known cognitive processing difficulties in PHIV+ youth.

## Abstract 2

Supplemental Digital Content is available in the text

## INTRODUCTION

The incidence of perinatally acquired human immunodeficiency virus (HIV) infection (PHIV+) in the United States has dropped dramatically over the past decade due to maternal use of combination antiretroviral therapy (cART) during pregnancy and delivery.^1^ The more widespread treatment of PHIV+ children with combination antiretroviral regimens has also resulted in substantial declines in morbidity and mortality.^2,3^ Although PHIV+ children are surviving longer with cART, they remain at risk for cognitive deficits, including poor executive functioning functioning, processing speed, attention, and visuospatial processing.^4,5^ Despite reconstituted immune and virologic status, HIV immunosuppression early in development is also associated with lower global cognitive performance,^6^ working memory,^7^ and processing speed.^8^ The pathogenesis of these neurocognitive deficits in PHIV+ youth is likely multifactorial, including the impact of early HIV infection, immune activation and suppression, and coinfections on the developing brain. Reports from both postmortem tissue and in-vivo imaging have revealed significant changes to cortical structures related to HIV infection in adults,^9,10^ and magnetic resonance imaging (MRI) studies have shown brain lesions, including white matter alterations in PHIV+ youth.^11–13^ However, employing additional neuroimaging techniques may help to determine the neurobiological effects of PHIV+.^13^ The current study aims to assess the relationship between markers of HIV severity and functional brain network connectivity in PHIV+ youth.

Resting-state functional connectivity MRI (rs-fcMRI) is used to assess the activity within and between brain networks when participants are not performing a specific task (e.g., laying awake with eyes closed in the scanner). Rs-fcMRI measures correlations of low-frequency blood oxygen-level-dependent (BOLD) signal fluctuations between a specific region-of-interest (ROI, e.g., a seed region) and all other brain voxels. Voxels in which the BOLD signal significantly correlates with the BOLD signal of a given seed region is considered “functionally connected.”^14,15^ Rs-fcMRI has revealed that the brain contains a number of large-scale networks, and that the default mode network (DMN) is most active at rest. The DMN primarily comprises the posterior cingulate cortex (PCC), medial prefrontal cortex (mPFC), lateral parietal cortices, and the inferior temporal lobes.^16–20^ As the brain develops, positive BOLD signal correlations are seen among DMN brain regions.^21–25^ By adulthood, regions within the DMN network show strong, positive BOLD signal correlations with one another, as well as strong, negative BOLD signal correlations between DMN and task-positive networks,^26^ such as the dorsal attention, fronto-parietal, and cingulo-opercular networks, during rest.^27^ Thus, with increased development, functional connectivity patterns are thought to include more positive within, and more negative between network correlations.

The current study examined how biological markers of HIV disease severity relate to DMN rs-fcMRI in PHIV+ youth. Greater HIV disease severity is reflected in a lower CD4 lymphocyte percentage (CD4%), and a higher plasma HIV ribonucleic acid (RNA) concentration. Given that altered DMN connectivity has been observed in HIV+ adults,^28^ we hypothesized that disease severity would relate to altered DMN connectivity. Specifically, we expected greater PHIV+ disease severity (i.e., higher peak HIV RNA and lower nadir CD4%) to predict within DMN connectivity pattern among PHIV+ youth (i.e., weaker positive BOLD signal correlations within DMN brain regions). Furthermore, we hypothesized that weaker within DMN connectivity would relate to the greater deficits in working memory and processing speed previously seen in PHIV+ youth.^6,7,29^

## METHODS

### Study Population

We recruited 40 PHIV+ youth, from Lurie Children's Hospital of Chicago, participating in the Adolescent Master Protocol study of the NIH Pediatrics HIV Acquired Immune Deficiency Syndrome (AIDS) Cohort Study (PHACS) network. Institutional review board approval at the participating site and Harvard School of Public Health was obtained. Parents or legal guardians and youth who were 18 or older provided written informed consent for research participation, adolescents provided assent.

### Disease Markers and Cognitive Functioning in PHIV+ youth

As previously described in more detail,^6^ Adolescent Master Protocol study visits were conducted semiannually through 2010 and annually thereafter. At each study visit, clinical diagnoses and laboratory results, including CD4% and plasma HIV RNA concentration (viral load), were abstracted from medical charts. At study entry, the lowest known CD4% (“nadir CD4%”) and highest known HIV viral load (“peak RNA”) prior to entry were collected. Lower nadir CD4% reflects poorer immune health, while higher peak RNA reflects worse virologic status. Diagnoses of encephalopathy were previously reviewed and confirmed by a pediatric neurologist.

The Wechsler Intelligence Scale for Children, Fourth Edition (6–16 years), and the Wechsler Adult Intelligence Scale, Fourth Edition for 17+^30,31^ were used to evaluate working memory index and processing speed index in PHIV+ youth. The majority of the subjects completed the age-appropriate Wechsler test within 1 year of the MRI scan (n = 38), with 77.5% being tested within 3 months of brain imaging (n = 31, mean ± standard deviation: 0.07 ± 0.57 years, range: 0–2.7 years).

### Image Acquisition

Images were collected on a single 3.0 Tesla Siemens Magnetom Tim Trio scanner (Siemens Medical Solutions, Erlangen, Germany) with a 12-channel head coil. A whole-brain structural T1 weighted magnetization prepared rapid gradient-echo image was acquired sagittally with a repetition time (TR) = 2170 ms, echo time (TE) = 4.33 ms, inversion time (TI) = 1100 ms, flip angle = 7°, acquisition matrix = 256 × 256, 192 slices, slice thickness = 1.1 mm. A 6-minute resting-state scan run was collected using a T2^∗^ weighted echo-planar imaging (EPI) sequence. EPI parameters were acquired axially with a TR = 2000 ms, TE = 30 ms, flip angle = 75°, field of view = 64 × 64, 33 slices, slice thickness = 4 mm with 4.99 mm gap, 180 repetitions. Participants were instructed to remain awake with their eyes closed during the scan.

### Image Processing and Analyses

Standard functional magnetic resonance imaging (fMRI) and rs-fcMRI preprocessing were performed using the Configurable Pipeline for the Analysis of Connectomes (version 0.3.3; http://fcon_1000.projects.nitrc.org/indi/cpac/index.html). This pipeline is an open source Nipype-based program that interfaces with FMRIB Software Library^32^ and Analysis of Functional NeuroImaging,^33^ and it has been outlined and used by the 1000 Functional Connectomes Project (www.nitrc.org/projects/fcon_1000/). FMRI preprocessing included EPI deobliquing, slice timing correction, motion correction, skull stripping, grand mean scaling, temporal band-pass filter (0.005 Hz < f < 0.1 Hz), spatial filtering (full width at half maximum = 6 mm), and removal of linear and quadratic trends in the data. A series of affine linear transformations were used to align the EPI to the anatomical image (6 degrees of freedom(dof)), and then the EPI and anatomical to standardized MNI152 stereotaxic space (12-dof). Regression analyses were used to regress out the 6 parameters obtained by motion correction, the average global signal regression (GSR), the ventricular signal averaged from the CSF ROI, and the white matter signal averaged from the white matter ROI. GSR is a composite measure that includes various sources of variation (e.g., respiration, cardiac signal, hardware stability, and magnetic field drifting). Arguments have been made both for^34–37^ and against^38,39^ using GSR. Because respiration and cardiac signals were not externally measured at the time of scan, and GSR reduces such nuisance variance in the fMRI signal,^35,37^ we chose to include it in our initial preprocessing stream to help increase tissue sensitivity.^34,36^ Initial analyses including the GSR during preprocessing resulted in 12 significant clusters for peak RNA and 9 significant clusters for nadir CD4%. However, to ensure these relationships were not an artifact of GSR, we performed a post-hoc analysis that included examining if the relationships between peak RNA and nadir CD4% remained similar if GSR was not included during preprocessing (see supplemental material, http://links.lww.com/MD/A409). Specifically, non-GSR connectivity values were extracted from significant clusters and post-hoc regression analyses were used to determine if peak RNA and nadir CD4% remained significantly related to functional connectivity correlation coefficients. Given that one of the beneficial mathematical factors of using the GSR includes reducing nuisance variance in the fMRI signal, we expected that previous significant connectivity results’ would have larger (less significant) *P*-values due to increased variance. Thus, similar relationships were defined as those that continued to show at least a trend-level (*P* ≤ 0.09) relationship. Only clusters that were deemed similar were further examined and are presented below.

To further reduce the systematic effects of movement on functional connectivity,^40,41^ a temporal mask was generated to detect TRs and their surrounding frames (1-back and 2-forward) that had both high-motion (i.e., framewise displacement >0.5 mm) and large BOLD amplitude changes (i.e., DVARS > 0.5%). This temporal mask was used to regress out additional artifacts due to motion prior to seed-based fcMRI analyses. After detection and removal of motion, the study sample was limited to only those with at least 5 minutes of “clean” resting-state data (final n = 31, Table 1) necessary to estimate functional connectivity networks.^40,41^

**TABLE 1:**
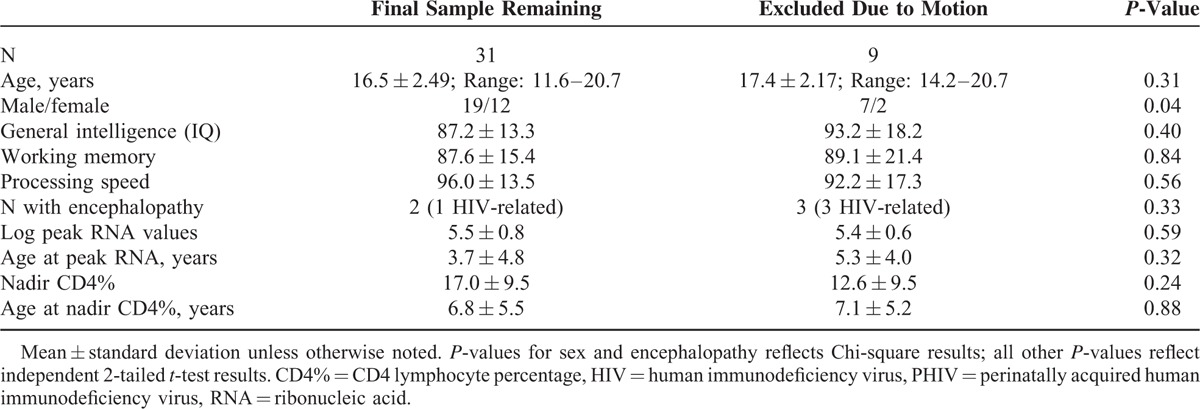
Sociodemographic, HIV Disease Severity, and Cognitive Functioning Measures Among PHIV+ Youth Assessed for Default Mode Network Connectivity

DMN seed regions consisted of five 12 mm spheres centered upon previously published DMN coordinates, including: PCC (0, −52, 27), mPFC (−1, 54, 27), bilateral lateral parietal cortices (left: −46, −66, 30; right: 49, −63, 33), and bilateral inferior temporal lobes (left: −61, −24, −9; right: 58, −24, −9).^27^ These seed regions were converted to MNI152 space prior to extracting the seed region BOLD time series data and computing voxelwise BOLD correlation maps. Lastly, a Fisher z transformation was applied to the correlation coefficients to improve normality for subsequent analyses.

### Statistical Analyses

Data were examined for normality, and when appropriate, transformations or nonparametric statistics were used. Specifically, a log (base 10) transformation was applied to peak viral load as well as age of peak viral load to more closely approximate normality, whereas nadir CD4% and age of nadir CD4% were approximately normal and were not transformed. Multiple regression analyses were performed to examine the relationship between past HIV disease severity markers (peak RNA or nadir CD4%) and cognition (working memory index and processing speed index), while controlling for age at peak RNA or nadir CD4%. Using Analysis of Functional NeuroImaging software, separate multiple regressions were performed for each DMN seed region with each HIV disease severity marker (peak RNA or nadir CD4%) as the primary independent variable, while controlling for scan age, biomarker age (e.g., age at peak viral load or age at nadir CD4%), and biomarker age-by-biomarker interaction. To correct for multiple comparisons, both a voxelwise and cluster-size threshold were applied. Specifically, a Monte Carlo simulation was performed, with results for all analyses requiring a voxel threshold of *P* < 0.01 and 1168 microliters (mm^3^) in volume (cluster probability threshold of *P* < 0.01) necessary for cluster significance. Significant clusters were identified by the peak location using the Harvard-Oxford Cortical Structural Atlas in FMRIB Software Library and the resting-state pediatric imaging template.^42^

## RESULTS

### Study Population

Forty youth with PHIV+ (mean age at scan = 16.7 ± 0.38 years; range 11.6–20.7 years old); 70% were African-American, and 12.5% were Hispanic) were enrolled. Seven boys (7/26 = 26.9%) and 2 girls (2/14 = 14.3%) were excluded from the study due to having less than 5 minutes of clean resting-state data necessary to estimate functional connectivity networks (demographics are shown in Table 1). Thus, the final sample of the analyses included 31 youth (19 boys and 12 girls). There were no significant differences between participants who were included and those excluded, except that boys (n = 7) were more often excluded due to motion compared to girls (n = 2). Importantly, for the final sample of 31, motion (as defined by mean framewise displacement) did not relate to the independent variables of interest or covariates (*r* ≤ −0.22, *P* > 0.24 for all measures). In addition, the percent of TRs excluded also did not relate to the independent variables of interest or covariates (*r* ≤ 0.25, *P* > 0.18 for all measures). Thus, motion parameters were not included in subsequent analyses.

### Peak RNA

**FIGURE 1:**
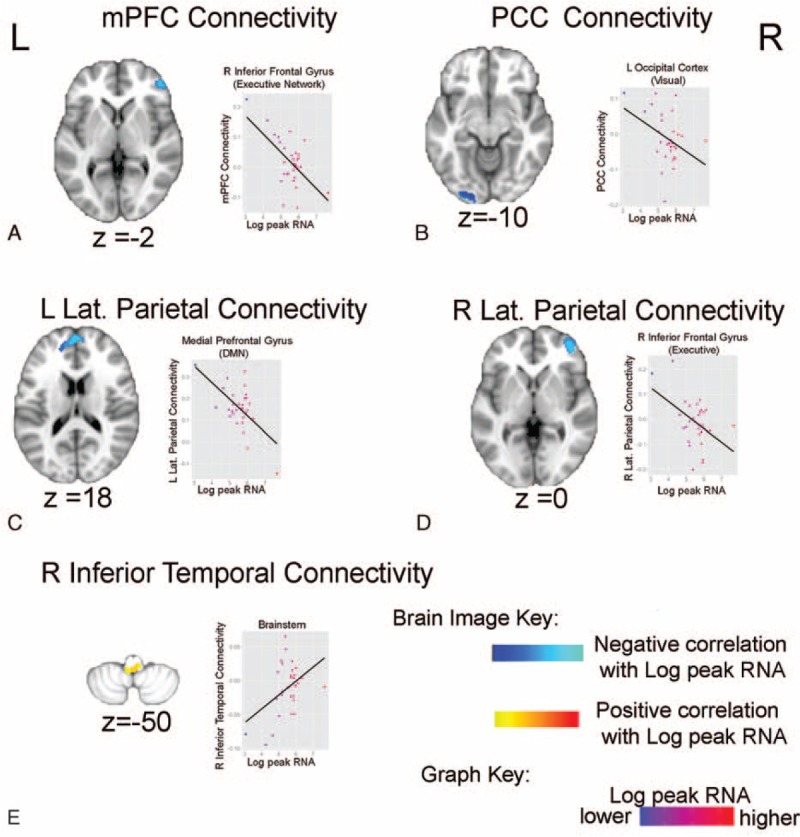
Peak HIV RNA levels and default mode functional connectivity. Brain images: blue reflects a negative correlation with peak HIV RNA levels; red-yellow reflects a positive correlation with peak HIV RNA levels. (A) Brain regions whose connectivity with the mPFC seed region relate to peak HIV RNA. (B) Brain regions whose connectivity with the PCC seed region relate to peak HIV RNA. (C) Brain regions whose connectivity with the left lateral parietal seed region relate to peak HIV RNA. (D) Brain regions whose connectivity with the right lateral parietal seed region relate to peak HIV RNA. (E) Brain regions whose connectivity with the right inferior temporal seed region relate to peak HIV RNA. HIV = human immunodeficiency virus, L = left, mPFC = medial prefrontal cortex, PCC = posterior cingulate cortex, R = right, RNA = ribonucleic acid.

Peak RNA levels were observed to relate to mPFC, PCC, right and left lateral parietal, and right inferior temporal connectivity (Table 2). Higher peak RNA levels related to larger negative BOLD correlations between the mPFC seed region and the right inferior frontal gyrus (executive network) (Figure 1A). Higher peak RNA levels also related to larger negative BOLD correlations between the PCC seed region and the left occipital cortex (visual network) (Figure 1B). For the lateral parietal seed regions, higher peak RNA levels also related to larger negative BOLD correlations between the left parietal seed region and cluster in the medial frontal gyrus (DMN) (Figure 1C), as well as the BOLD correlations between the right seed region and the R inferior frontal gyrus (executive network) (Figure 1D). For the right inferior seed region, higher peak RNA levels related to a positive BOLD correlation with the brainstem (Figure 1E).

**TABLE 2:**
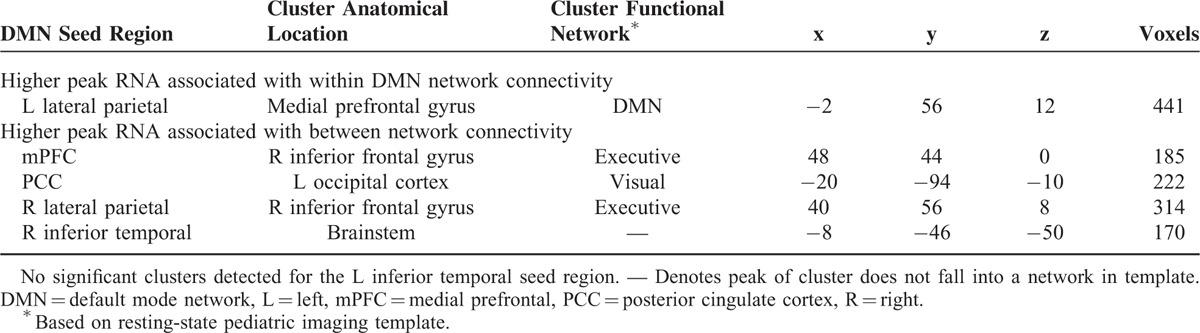
Significant Relationships Between Peak HIV RNA Levels and DMN Functional Connectivity (Controlling at Age of Peak RNA and Age at Scan)

**FIGURE 2:**
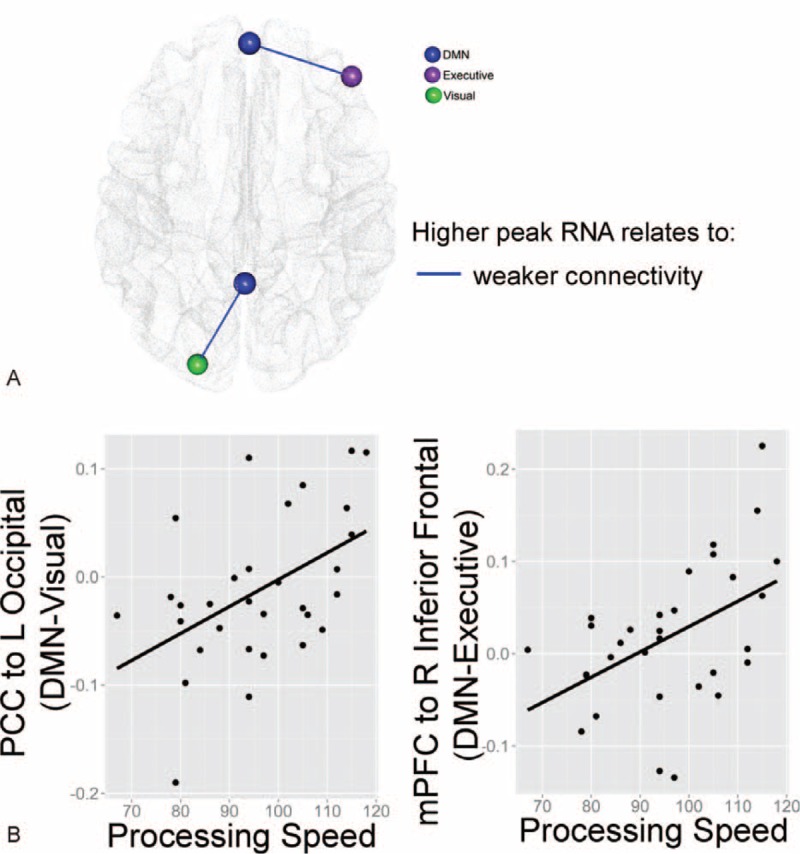
Peak RNA, DMN functional connectivity, and processing speed. (A) Visual depiction of associations (overlaid on top of a semitransparent axial brain) between peak RNA and connectivity that were found to relate to processing speed. Sphere color represents regions functional network based on the resting-state pediatric imaging template. (B) Graphs showing PCC to left occipital connectivity and mPFC to right inferior frontal connectivity positively relates to processing speed scores. Y-axes reflect resting-state connectivity (positive and negative correlations). DMN = default mode network, L = left, mPFC = medial prefrontal cortex, PCC = posterior cingulate cortex, R = right, RNA = ribonucleic acid.

After controlling for age of peak RNA, peak RNA levels were significantly associated with processing speed (β = −7.54, SE = 2.26, *P* = 0.002) but not working memory (β = −5.66, SE = 2.86, *P* = 0.058). Exploratory post-hoc analyses were performed to determine if significant log peak RNA DMN connectivity findings related to processing speed ability. Specifically, BOLD correlation coefficients of each significant peak RNA cluster were extracted for each subject. Separate multiple regression analyses were performed to examine the relationship between peak RNA-related DMN BOLD correlation coefficients and processing speed indices, while controlling for age of scan. Results showed that DMN connectivity of the mPFC and PCC significantly predicted processing speed ability (Table 3, Figure 2A). Specifically, stronger positive BOLD correlations between the mPFC to right inferior frontal gyrus (DMN-Executive connectivity) and stronger positive BOLD correlations between the PCC to the left occipital cortex (DMN-Visual connectivity) predicted better processing speed scores (Figure 2B).

**TABLE 3:**
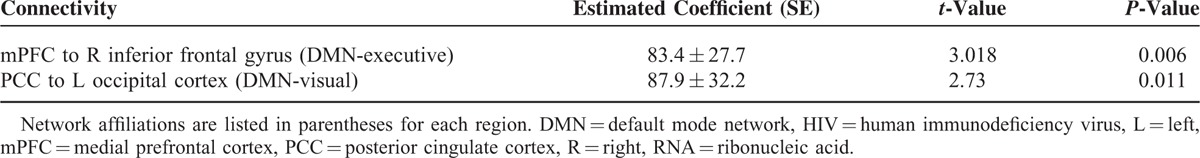
Significant Relationships Between Significant Peak HIV RNA Connectivity Clusters and Processing Speed

### Nadir CD4%

**FIGURE 3:**
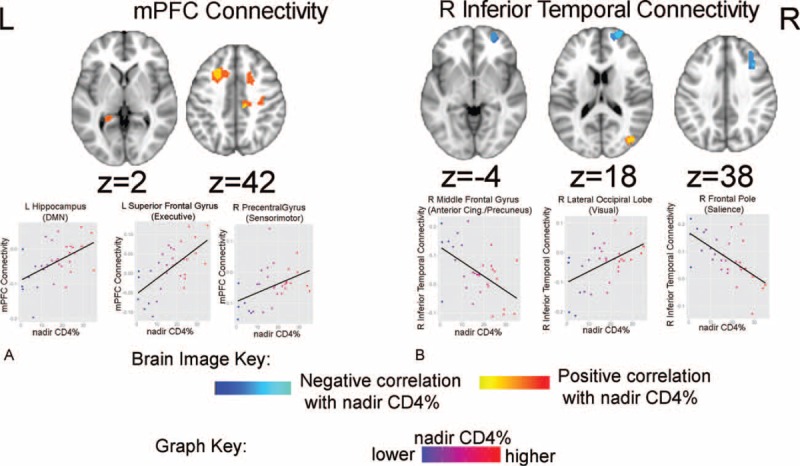
Nadir CD4% and default mode functional connectivity. Brain images: blue reflects a negative correlation with nadir CD4% levels; red-yellow reflects a positive correlation with nadir CD4% levels. (A) Brain regions whose connectivity with the mPFC seed region relate to nadir CD4% levels. (B) Brain regions whose connectivity with the right inferior temporal seed region relate to nadir CD4% levels. CD4% = CD4 lymphocyte percentage, L = left, mPFC = medial prefrontal cortex, R = right.

Nadir CD4% was found to relate to mPFC and right inferior temporal connectivity (Table 4). Specifically, lower nadir CD4% levels related to larger negative BOLD correlations between the mPFC and left superior frontal gyrus (executive network), left hippocampus (DMN), and the right precentral gyrus (sensorimotor network) (Figure 3A). Lower nadir CD4% was also related to larger positive BOLD correlations between the right inferior temporal seed region and the right frontal pole (salience network) and the right middle frontal gyrus (anterior cingulate/precuneus network), as well as larger negative BOLD correlations between the right inferior temporal seed region and the right lateral occipital lobe (visual network) (Figure 3B). Nadir CD4% was not a significant predictor of working memory or processing speed when controlling for age at nadir CD4%.

**TABLE 4:**
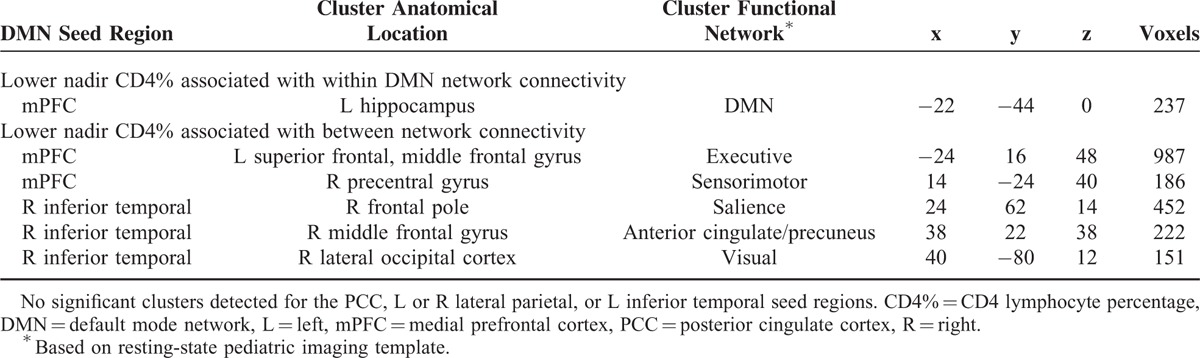
Significant Relationships Between Nadir CD4% and DMN Functional Connectivity (Controlling for Age of Nadir CD4 and Age of Scan)

## DISCUSSION

**FIGURE 4:**
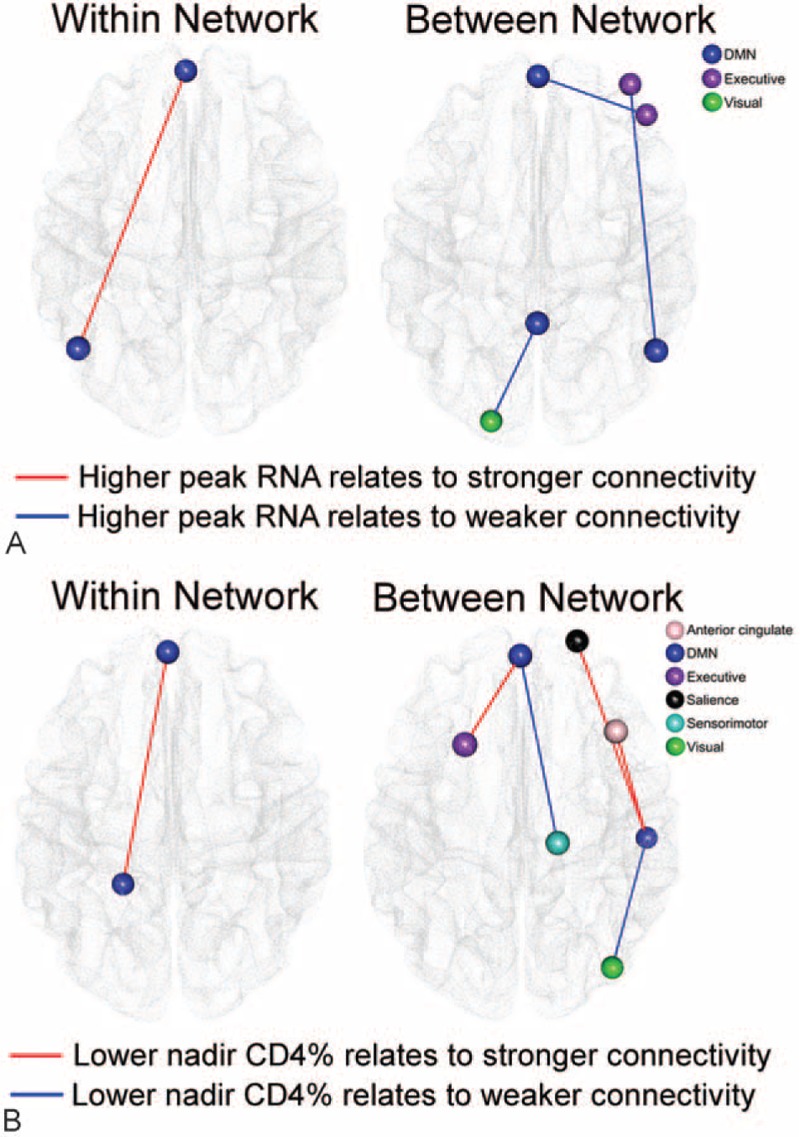
Visual depiction of significant associations between disease severity markers and default mode network (DMN) connectivity overlaid on top of a semitransparent axial brain. Sphere color represents regions’ functional network based on the resting-state pediatric imaging template.

The current study is the first to demonstrate that biological markers of disease severity significantly relate to patterns of DMN connectivity in PHIV+ youth. Based on altered DMN connectivity observed in HIV+ adults,^28^ we hypothesized that greater PHIV+ disease severity (i.e., higher peak HIV RNA and lower nadir CD4%) would predict alterations in the within DMN connectivity pattern among PHIV+ youth (i.e., weaker positive BOLD signal correlations within DMN brain regions). However, as summarized in Figure 4, our results show that disease severity related to both increases and decreases in BOLD signal correlations for both within- and between-network DMN connectivity. These findings suggest that HIV severity may lead to substantial reorganization of the DMN and its connectivity with task-positive and sensory-related networks. Furthermore, this reorganization of within and between DMN connectivity patterns was associated with better processing speed ability in this sample. Thus, peak HIV RNA-related alterations in DMN connectivity coincides with known domains of cognitive risk in children with perinatally acquired HIV.

Our findings suggest HIV severity and immune function relate to altered DMN within- and between-network connectivity in PHIV+ youth. In healthy individuals, DMN regions show positive within-network BOLD correlations with one another, but negative between-network BOLD correlations with other brain regions at-rest, including “task-positive” networks (dorsal attention, fronto-parietal, cingulo-opercular, etc.).^26,27^ In studies with typically developing healthy children, large-scale networks show a number of age-related changes, with less within-network, but greater between-network connectivity reflective of a pattern of connectivity occurring earlier in development.^22,43,44^ Interestingly, in this study, we found that PHIV+ youth with more advanced HIV disease severity manifest some of these markers of a “less mature” DMN network. For example, weaker DMN to DMN within-network connectivity (e.g., peak RNA: left lateral parietal seed region and medial prefrontal gyrus; nadir CD4%: mPFC and the left hippocampus), and stronger connectivity between the DMN and other functional networks (e.g., visual, sensorimotor, executive, salience, anterior cingulate, and precuneus). These patterns in PHIV+ children are congruent with previous findings in HIV+ older adults that found less positive correlations between a DMN seed region (right lateral parietal) and other DMN brain regions,^28^ as well as decreased resting-state oscillations in the posterior cingulate DMN region using magnetoencephalography in HIV+ elderly compared to age-matched adults.^45^ Similarly, findings of positive BOLD correlations between the DMN seed regions, such as the attention network regions in HIV+ older adults suggest that brain networks are less separate at-rest compared to unaffected individuals.

Previous rs-fcMRI has shown disruptions in the lateral occipital cortex network in HIV+ adults with infections within the past year^46^ while other large-scale networks, including the DMN, were not affected compared to age-matched controls. In contrast, we found both peak RNA and nadir CD4% to relate to an altered connectivity pattern between a number of the DMN seed regions, including the connectivity between the DMN and lateral occipital regions. Although functional connectivity does not necessarily reflect structural connectivity, these findings are consistent with bilateral posterior corpus callosum white matter atrophy recently reported in perinatally HIV-infected youth.^47^ Moreover, in our study, lower nadir CD4% was related to less negative connectivity between the mPFC of the DMN and the hippocampus. Smaller hippocampal volumes have been reported in HIV+ adults,^48,49^ which have also shown to relate to disease severity (i.e., nadir CD4).^50^ Furthermore, HIV+ transgenic mouse models show extensive changes in hippocampal synaptic physiology.^51^ Given that the hippocampus plays a crucial role in learning and memory,^52^ further research is needed to determine how mPFC and hippocampus connectivity in PHIV+ might relate to other cognitive domains such as long-term memory. Together, current findings suggest that HIV disease severity may lead to prominent changes in the visual network and hippocampal connectivity at rest.

Of interest, the peak RNA and nadir CD4% had different relationships with DMN connectivity in our PHIV+ youth (Figure 4), suggesting that a history of advanced immune suppression and higher peak HIV RNA levels may have varying effects on brain function.^4^ Although progression of HIV infection generally leads to a subsequent drop in CD4 cell count, CD4 count and HIV viral load do not always directly relate to one another. Furthermore, brain function in PHIV+ youth may be impacted by lifelong HIV infection, intermittent, or ongoing viral replication in the central nervous system, ongoing neuroinflammation, irreversible central nervous system damage prior to cART initiation, and/or neurotoxic effects of cART, all of which may contribute to the varying effects of CD4% (immune status) compared to viral load (viral replication). Also of interest was that peak RNA-related patterns of DMN connectivity were associated with cognition in the current study. Specifically, mPFC connectivity with the right inferior frontal gyrus, as well as PCC connectivity with the left occipital cortex related to process speed ability. Although patterns of functional connectivity vary during rest and goal-oriented tasks,^53^ it is feasible that HIV-related changes in the DMN connections with these prefrontal and visual regions may contribute to differences in processing of visual stimuli, attention, and/or cognition that could ultimately affect processing speed in PHIV+ youth. Although cause and effect of these altered patterns cannot be determined by this cross-sectional study, longitudinal and task-based neuroimaging studies of PHIV+ children are warranted to determine if these altered connections remain stable over time and across imaging paradigms (rest- versus task-based).

Although this is the first report of relationships between disease markers and DMN connectivity in PHIV+ youth, a number of limitations should be noted. First, there is not yet consensus in the rs-fcMRI field in terms of using GSR during preprocessing.^34–39^ In the current study, we have chosen to use GSR during preprocessing to help reduce the variance in our fMRI signal due to respiration, cardiac signal, hardware, and magnetic field stability.^34–37^ To verify directionality of our results, we also examined our results excluding the GSR and only reported on those that remained similar using both approaches (supplemental material, http://links.lww.com/MD/A409). Thus, while some controversy continues to exist in the field about GSR, we feel confident that the results and interpretations of the current study are valid. Secondly, in order to put the current findings in context, we described network locations based on networks defined by independent component analysis. It is feasible that variability in network topography could exist between the healthy, HIV-negative atlas, and the current HIV+ pilot study participants. Future studies that include collecting HIV+ and HIV− cohorts within the same study and on the same scanner are necessary to determine if independent component analysis networks are influenced by perinatal exposure to HIV. Along the same lines, it is not known how immune system markers are related to DMN connectivity in typically developing youth. Third, the majority of the PHIV+ youth in the current study were on cART, making it impossible to disentangle the contributions of HIV disease status and cART to the current findings. Thus, future research with larger sample sizes is needed to clarify the effects of the HIV infection compared to combination drug therapies on brain organization and function. A number of these limitations may be best addressed by designing future studies to include 3 groups, including a healthy HIV-unexposed control group, a perinatal HIV-exposed but uninfected group, and a sample of PHIV+ youth. Lastly, in order to elucidate whether relationships between disease markers and connectivity are dynamic or remain static with age, longitudinal investigations are necessary.

In conclusion, this is the first report of relationships between disease markers and DMN connectivity in PHIV+ youth, and highlights significant alterations in functional networks based on disease severity. The present study, together with previous findings with structural connectivity,^12^ suggests that altered patterns of brain connectivity are affected by HIV+, and these neurobiological alterations may contribute to risk for cognitive problems among PHIV+ youth.
